# Brief Brain Tissue Donation Community Presentations Increase Knowledge, Decrease Misinformation and Improve Brain Donation Acceptability

**DOI:** 10.1002/alz70860_106940

**Published:** 2025-12-23

**Authors:** Allison M Caban‐Holt, Michael L Cuccaro, Ashley Gregory, Brianna Eley, Tina Duke, Jenny Chavez, Devon Jones, Katherine Racy, Shawnta Lloyd, Takiyah D. Starks, Larry D Adams, Jonathan L Haines, Christiane Reitz, Jeffery M Vance, Margaret Pericak‐Vance, Goldie S Byrd

**Affiliations:** ^1^ Wake Forest University School of Medicine, Winston‐Salem, NC, USA; ^2^ John P. Hussman Institute for Human Genomics, University of Miami Miller School of Medicine, Miami, FL, USA; ^3^ Maya Angelou Center for Health Equity, Wake Forest University School of Medicine, Winston‐Salem, NC, USA; ^4^ Gertrude H. Sergievsky Center, Taub Institute for Research on the Aging Brain, Departments of Neurology, Psychiatry, and Epidemiology, College of Physicians and Surgeons, Columbia University, New York, NY, USA; ^5^ Cleveland Institute for Computational Biology, Case Western Reserve University, Cleveland, OH, USA; ^6^ Department of Population and Quantitative Health Sciences, School of Medicine, Case Western Reserve University, Cleveland, OH, USA; ^7^ Department of Population and Quantitative Health Sciences, Institute for Computational Biology, Case Western Reserve University, Cleveland, OH, USA; ^8^ University of Miami Miller School of Medicine, Miami, FL, USA

## Abstract

**Background:**

Black∖African Americans (B∖AA) are underrepresented in Alzheimer's disease (AD) brain tissue donation (BTD) research. Availability of a diverse ancestral collection of brain tissue is critical for functional genomic studies on the pathway to prevention and treatment solutions. An educational presentation was developed to increase knowledge about BTD procedures, and correct BTD misinformation for B∖AA community members. Focus groups were conducted to determine whether the presentation was acceptable, understandable, and conveyed the intended factual information to attendees.

**Method:**

Knowledge surveys were administered before and after BTD Education PowerPoint presentations to focus groups in Florida, New York, North Carolina, and Ohio between October and December 2024. The survey was comprised of 4 self‐report items,12 BTD, and 2 AD knowledge items based upon topics covered in the presentation.

**Result:**

B∖AAs participating in focus groups (*n* = 21) were primarily female (87.5%), average age of 69.8 years, and average education of 13.4 years. At baseline, 90.5% of participants reported “very little” knowledge about BTD, whereas after the presentation only 4.8% reported little knowledge and instead 76.2% reported “much∖very much” knowledge after the presentation. In terms of attitudes toward brain donation, pre‐tests showed 42.9% reported being “not very likely∖unlikely” to donate their brain to research, while at post‐test 57.14% reported being “likely∖very likely” to donate their brain.

The group averaged 71.9% correct on knowledge items on the pre‐test and 85.7% on the post‐test; an increase of 13.8% by attending the presentation. The survey item with the highest percentage of incorrect responses on both the pre‐test and post‐test was “Who is the first point of contact that should take place after a person who wants to donate brain tissue passes away?”, suggesting that clearer information needs to be conveyed regarding the steps followed after a brain donor's death.

**Conclusion:**

Overall, brief presentations were shown to be impactful tools for improving audience member knowledge of BTD procedures and increasing positive attitudes toward BTD in this B∖AA sample. This research team plans to provide BTD presentations and accompanying educational materials widely with a goal of enrolling 100 B∖AA into a BTD program in the next 24 months.

